# Active Moms: a feasibility study of a community-based and home-based physical activity intervention for low-income, ethnic-minority mothers

**DOI:** 10.1186/s40814-023-01378-z

**Published:** 2023-11-17

**Authors:** Wendy Miranda Arevalo, Brianna Isabel Caicedo, Guido G. Urizar, Jan Schroeder

**Affiliations:** 1 Department of Environmental Health Sciences, UCLA Fielding School of Public Health, Los Angeles, CA USA; 2 Department of Urban Planning, UCLA Luskin School of Public Affairs, Los Angeles, CA USA; 3 Department of Psychology, California State University, Long Beach, 1250 Bellflower Blvd, Long Beach, CA 90840-0901 USA; 4 Department of Kinesiology, California State University, Long Beach, Long Beach, CA USA

**Keywords:** Physical activity, Community, Home, Intervention, Ethnic minority, Mothers

## Abstract

**Background:**

Despite the health benefits of physical activity, increasing regular physical activity levels among low-income, ethnic-minority mothers has remained a significant challenge. Yet, few studies have examined the feasibility of implementing interventions to address physical activity barriers often experienced by this population.

**Methods:**

The current mixed-methods pilot study assessed the feasibility, impact, and acceptability of a 3-month community-based (CBI) and a home-based intervention (HBI) designed to improve physical activity and fitness levels, as well as psychosocial outcomes (self-efficacy and social support), among low-income, ethnic minority mothers. Mothers were randomized to either a 3-month CBI or HBI and completed pre- and post-intervention assessments of physical activity, fitness, self-efficacy, and social support. Intervention feasibility was assessed by tracking recruitment, retention, and adverse event rates, whereas intervention acceptability was assessed through post-intervention focus groups.

**Results:**

Although participant recruitment was lower than expected (30 vs. target of 52 mothers), retention and adverse event feasibility goals were met (> 60% retention rate, 0% adverse events). Mothers in both groups (CBI and HBI) showed significant improvements in their physical activity and fitness levels and short-term improvements in receiving social support for physical activity. However, only mothers in the CBI group showed improvements in their self-efficacy for physical activity. Mothers also reported both types of interventions (CBI and HBI) to be acceptable with minor modifications highlighted, including the potential for graduates of these programs to serve as group facilitators.

**Conclusions:**

Overall, the study protocol was feasible, impactful, and acceptable to low-income, ethnic minority mothers with modifications required before large-scale evaluation. (TRN: NCT05540509; 9/12/22; retrospectively registered; ClinicalTrials.org).

## Key messages regarding feasibility

- Few studies have examined the feasibility of implementing interventions of different modalities to address physical activity barriers often experienced by low-income, ethnic-minority mothers.
- The key feasibility findings of this study are that although the protocol to meet study recruitment goals were not met, retention goals and minimization of adverse events were feasible, mothers found both the CBI and HBI to be acceptable, and both interventions were effective in improving physical activity and fitness levels.
- Implications of study findings for the main study design are modifications to the recruitment protocol, including integrating mothers who are graduates of these programs to serve as recruitment coordinators and intervention group facilitators to better support low-income, ethnic minority mothers in their efforts to engage in physical activity.

## Introduction

It has been well established that engaging in physical activity (PA) leads to several positive health outcomes such as preventing cardiovascular disease, reducing symptoms of depression and anxiety, and lowering all-cause mortality rates [1]. Despite these health benefits, increasing regular PA levels, particularly among low-income ethnic-minority mothers, continues to be a significant challenge. Although current PA guidelines recommend that all US adults accumulate at least 150 min of moderate-intensity or 75 min of vigorous-intensity aerobic activity per week (e.g., running, walking), 2 or more days of muscle-strengthening exercises (e.g., lifting weights) per week, and 2–3 days of flexibility exercises (e.g., stretching) per week [2, 3], only 21% of women meet these public health recommendations, with mothers of young children being even less likely to meet these recommendations (< 11%) [4–6]. These low rates of PA may be due to competing childcare demands and responsibilities, financial strain related to childcare, and having a lack of social support or self-efficacy to exercise [5, 7–9]. Given these findings, feasibility studies are needed to explore what types of intervention approaches might best facilitate the adoption of PA in this population.

Interventions based on theories, such as Bandura’s Social Cognitive Theory [10], have been effective in increasing PA across various populations. Two specific components of this theory, reciprocal determinism and collective agency, have been identified as being particularly important for increasing PA. Reciprocal determinism focuses on how personal (e.g., self-efficacy) and environmental factors (e.g., social support) interact with one another to influence future behavior change (e.g., engaging in PA) [10]. Similarly, the concept of collective agency is important in understanding how individuals can affect their environment to enable changes in their behavior. Collective agency emphasizes a group’s shared belief to influence behavior change by working together, motivating one another, determining how to use their resources, and how to overcome obstacles to meet a common goal [11]. Both reciprocal determinism and collective agency can be affected by sociocultural factors, such as one’s socioeconomic status and family structure to either augment or impede behavior change efforts through their impact on one’s sense of efficacy and social support, which are two constructs that have been targeted by interventions to facilitate the adoption and maintenance of PA [11]. Self-efficacy, which emphasizes one’s confidence to engage in long-term behavior change [10], has been shown to be a strong predictor of PA among low-income, ethnic-minority mothers [5, 12, 13]. Similarly, PA interventions that emphasize social support (e.g., having an exercise partner, support from family and friends, receiving guidance through classes and print-based or digital-based materials) or that utilize community-based settings (e.g., parks, churches, medical centers) have been shown to be strong motivating factors in increasing PA levels, especially among mothers [5, 12, 13]. Specifically, having the support of one’s spouse in sharing childcare/household responsibilities has been shown to increase mothers’ engagement in PA [5, 14]. However, for low-income ethnic minority mothers, qualitative studies reveal that lack of childcare, lack of support from others, and lack of confidence are common barriers to engaging in PA [15], highlighting the need to design and test different PA intervention modalities for this population.

Two common modalities used for promoting PA adoption and maintenance are community-based interventions (CBIs) and home-based interventions (HBIs). CBIs are typically led by trained facilitators that teach group-based PA classes in a community setting and have varied in their structure to enhance self-efficacy for PA [16]. Few studies have examined the efficacy of CBIs among mothers, with one study demonstrating that mothers who participated in a CBI that transitioned from a 4-week group-based to a 4-week home-based format showed significant improvements in PA during the group-based phase and maintained these changes during the home-based phase [17]. One other CBI study demonstrated that including a social support component (e.g., having an exercise partner) was particularly effective in increasing PA levels up to 6 months post-intervention among low-income Latina mothers [18]. In contrast to CBIs, HBIs have traditionally focused on sending participants educational materials (printed or digital) about setting behavioral goals and overcoming barriers to PA, which places responsibility on participants to review this information and increase their PA on their own [19–21]. Recent studies have shown HBIs that provide practical home exercises to be particularly effective in increasing self-efficacy and PA among low-income, ethnic-minority populations up to 12-months post-intervention [19–21].

Cumulatively, these findings highlight the need to document low-income, ethnic-minority mothers’ experiences in CBIs and HBIs to better understand how to improve the structure and content of these PA intervention modalities in this population. Qualitative studies have highlighted low-income mothers’ hesitations to participate in PA interventions due to competing time demands and responsibilities, as well as lack of efficacy and support to meet PA goals [14]. Few studies have examined how different types of support affect the adoption and maintenance of PA among low-income ethnic minority mothers over time [22]. Therefore, mixed methods studies are needed to explore the feasibility of PA interventions in addressing potential barriers to PA participation, mechanisms for PA increases, and areas for improvement in intervention design and delivery in this population.

### Present study

The current mixed-methods pilot study assessed the feasibility of delivering a study protocol of a 3-month CBI and a HBI among low-income, ethnic minority mothers in terms of recruitment, retention, and participant safety. This study also explored the impact that the CBI and HBI had on PA and fitness levels, self-efficacy, and social support in this population. To assess intervention acceptability, post-intervention focus groups were conducted to document mothers’ experiences while participating in their respective PA programs by identifying PA barriers and facilitators, as well as recommendations for program improvements.

## Methods

### Participants

Mothers were recruited between 2012 and 2013 via brochures that were distributed to local community health organizations and school districts in southern California that serve a predominantly low-income population (38% below poverty level) [23]. Mothers interested in the study called the research team to learn more information about the study purpose, eligibility, and requirements. Eligibility criteria included being 18 years of age or older, a mother of a young child (≤ 10 years of age; criteria based on a study showing parents with children in this age group being at highest risk for inactivity) [24], fluent in Spanish or English, medically cleared by a doctor or by a medical history screener (AAPQ; administered by research staff) [25] to engage in moderate-intensity PA, and sedentary (e.g., not engaging in 90 min or more of moderate or vigorous PA per week). Participants could not be currently pregnant.

### Procedures

All study procedures were approved by the Institutional Review Board at California State University, Long Beach (ClinicalTrials.gov TRN: NCT05540509). Eligible mothers who wanted to participate in the study were scheduled for an in-person visit (at a local community center where the CBI would take place) to sign a consent form and to complete a 1-h baseline assessment. This assessment was administered by trained research staff in Spanish or English to determine baseline fitness levels (i.e., cardiorespiratory fitness, muscular endurance and strength, flexibility) and psychosocial functioning (i.e., self-efficacy and social support). Afterwards, mothers received a take-home kit with instructions on how to self-report their PA levels (i.e., Check and Line Questionnaire; described below) and wear a Fitbit activity monitor over three consecutive days (two weekdays and one weekend day). The Fitbit display was wrapped with dark masking tape so that mothers’ baseline PA levels would not be influenced by the feedback provided by the Fitbit. After completing their baseline assessments, mothers met with research staff at the same community center to turn in their take-home kit materials and were then randomized to either the 3-month CBI or HBI group using a prospective, convergent mixed-methods, parallel assignment, randomized group design. Randomization was computer-generated, with group allocation concealed by opaque, sequentially numbered sealed envelopes (research staff were blinded to group assignment) to prevent selection bias. A 2:1 (CBI to HBI group) randomization ratio was used to oversample for the CBI group to account for expected differences in class attendance within this group. Group facilitators documented the number of classes attended by each mother in the CBI group. Follow-up PA, fitness, and psychosocial assessments were conducted at the 1-, 2-, and 3-month time points with mothers receiving a $30 gift card for completing the assessment at each time point (total of $120 in gift cards). After being assigned to their randomization group, participants met with a research staff member for a one-on-one introductory session to review safety guidelines for initiating their PA program and to set personal goals based on national PA recommendations and the mothers’ current PA level. At the 3-month post-intervention time point, participants were invited to participate in a focus group to assess their experiences in their respective programs and to gather program feedback.

### Randomization groups

#### Community-based intervention (CBI)

**Fig. 1:**
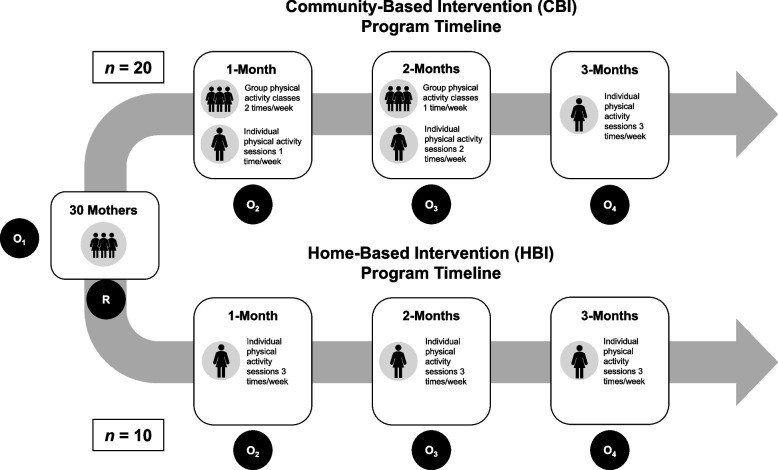
Study timeline by randomization group. Key: R = point in which the mothers were randomized into either the CBI or HBI; O_1_ = baseline assessments completed; O_2-4_ = follow-up observations/assessments completed (physical activity assessment, fitness assessment, and psychosocial assessment)

Mothers randomized to the CBI group participated in a 3-month program called Active Moms (Mamás Activas) aimed at supporting mothers’ goals to initiate and maintain PA through community resources. This program consisted of 12 group exercise sessions over a 3-month period that were offered in Spanish and English (the back translation and centering method was used to translate all intervention materials to Spanish) [26] and were taught by three masters-level group facilitators who were trained by a certified kinesiologist. The CBI group was offered to three separate cohorts (once in Spanish and twice in English) to groups of five to eight mothers in each cohort. The group exercise sessions were held at a local church near the neighborhoods where mothers were recruited from and were offered during the day to accommodate the operating hours of the church. Each 2-h session consisted of 90 min of flexibility, cardiovascular, and strength exercises to meet PA recommendations and 30 min of group discussions that focused on topics related to the adoption and maintenance of PA (see Table 1 for course content). For each session, mothers were given PA goals and coping resources to practice at home (e.g., identifying motivators and support for exercise) and were asked to record their experiences on an activity log that was collected and discussed during the next class session. Course content was taught from a detailed training manual [27] and was based on concepts and strategies from social cognitive theory that have been effective in increasing PA and fitness levels among mothers [9, 10, 17]. Over the course of the 3 months, group exercise sessions gradually decreased and individual exercises increased to enhance mothers’ confidence in maintaining long-term exercise gains on their own (see Fig. 1). In the first month, mothers attended group exercise sessions twice per week (total of eight group sessions) and exercised on their own once per week. In the second month, mothers attended group exercise sessions once per week (total of four group sessions) and exercised on their own twice per week. In the third month, mothers exercised on their own three times per week.

**Table 1:** Topics for CBI classes and HBI printed materials

Class	Class topics	CBI class description	HBI material description
1st month			
1	Introduction to Active Moms and Exercise Safety	Mothers were introduced to Active Moms and went over class contracts, guidelines, and PA safety (e.g., form, warm-ups, etc.).	Mothers were introduced to Active Moms and went over class contracts, guidelines, and PA safety (e.g., form, warm-ups, etc.)
2	Goal Setting	Mothers set SMART goals including short- and long-term goals.	Mothers set short- and long-term goals and discussed their current PA levels.
3	Motivation to Exercise	Mothers reflected their motivation for PA. Inspirational quotes were shared in class.	Mothers reflected on their likes and dislikes of PA, changes in PA, and any goals achieved.
4	Time Management	Mothers were taught strategies to improve time management and were given a time management log.	Mothers were given examples of active choices to increase minutes of PA and a time management log.
5	Problem Solving	Mothers were introduced to the “problem-solving loop” and were presented with hypothetical scenarios to use their problem-solving skills.	Mothers were introduced to the 4-step problem solving plan and asked to make a positive action plan.
6	Finding the Exercise That’s Right for You	Mothers were introduced to different types of PA and listed PA they would like to try on their own.	Mothers were given a list of ways to prevent boredom and a description of how to improve their aerobic fitness.
7	Community Resources	Mothers presented their PhotoVoice pictures on what resources in their community serve as PA facilitators.	Mothers were given a list of Long Beach Local Community Resources along with contact information.
8	Finding Support	Different types of social support and benefits of social support for meeting PA goals were discussed. Mothers were paired to discuss ways of exercising more.	Mothers were given examples of ways to explain to their peers of the support they needed when attempting to engage in PA and how to better help themselves engage in PA.
2nd month			
9	Working Out with the Kids	Mothers discussed being a role model for their children and different PA that can be done with their children. Mothers were then paired to discuss current and future PA with their children based on their children’s age and capabilities.	Mothers were given a handout which explained ways they could think outside of the box and create schedules with their children to help engage make PA a family activity.
10	Stress Management & Mindfulness	Mothers were taught the positive effects of PA on stress and engaged in a mindfulness walk.	Mothers were asked to list things that made them stressed, provided with examples of ways to prevent stress, and make an action plan.
11	Exercise Maintenance	Mothers discussed strategies to maintain their exercise gains and reflected on their personal preferences to exercising.	Mothers were given a handout which described ways to start an exercise program and how to stay committed to the program.
12	Falling Off the Wagon	Mothers discussed slips/lapses to PA routines and strategies to overcome these. Mothers were presented with hypothetical scenarios where they were asked to give advice to someone on how to maintain their exercise routine.	Mothers were asked to review their progress and ways to handle instances in which they experience slips or lapses in their PA routines.

#### Home-based intervention (HBI)

Mothers randomized to the HBI group participated in a 3-month program where they were given print-based materials (offered in Spanish or English) at each monthly assessment time point. The print-based materials provided information on multiple cardiovascular, strength training, and flexibility exercises that they could do at home (see Table 1 for more details). Similar to the CBI group, the HBI group received information on building social support and reducing PA barriers that was based on materials from the Diabetes Prevention Program [28]. Participants were also given activity logs to record their PA each week, which was collected by research staff at each monthly assessment time point to assess how participants were progressing with their PA goals. Mothers exercised on their own for 3 months with the goal of meeting national PA guidelines [2, 3].

### Measures

#### Sociodemographics

A sociodemographic questionnaire assessed for maternal characteristics such as age, number of children, total years of education, annual household income, and marital status.

#### Study feasibility

To determine the feasibility of the study protocol and the CBI/HBI interventions, information on study recruitment (number of mothers screened for eligibility, deemed eligible, and number consented), retention (number of participants completing baseline and follow-up assessments), and participant safety (number of health-related adverse events) were recorded by research staff.

#### Physical activity

Self-reported PA was assessed using the Check and Line Questionnaire (CALQ) [29]. The CALQ measures the type (e.g., walking, housework), intensity (moderate or vigorous), and duration (minutes) of PA engaged in each day. The duration of moderate/vigorous-intensity PA (excluding housework) per day was examined, with higher numbers representing greater average minutes of moderate/vigorous-intensity PA completed/day over the 3-day assessment period (*α range* = 0.84–0.95). The CALQ has been shown to be a reliable, valid, and effective measure of self-reported PA in ethnic-minority women, particularly when paired with an accelerometer [29]. Objective PA was assessed using the Fitbit One (2009 version), which is an accelerometer worn on the hip (clipped to a participant’s clothing at the waist). For the current study, very active minutes were examined (measure of basal metabolic rate/minute). Activity data was synced to an online database and was averaged across the three days that the Fitbit was worn, with greater number of very active minutes indicating greater daily moderate/vigorous-intensity PA levels. The Fitbit has been shown to be a reliable accelerometer and a valid activity tracker [30, 31].

#### Fitness

Cardiorespiratory fitness was assessed via predicted relative maximum oxygen consumption, also known as relative VO_2 max_ (ml/kg/min) after participants completed a timed mile walk [3, 32]. Their time was used to calculate the estimated relative VO_2 max_ using the following formula: *VO*_*2 max*_ = *132.85 − (0.0769* × *weight [lbs.]) − (0.3877* × *age)* + *(6.315* × *0 [0* = *Woman]) − (3.2649* × *time) − (0.1565 heart rate)*, with a greater relative VO_2 max_ being an indicator of greater cardiorespiratory fitness. Muscle endurance and strength were assessed by the number of modified push-ups completed in correct form (i.e., being in a bent-knee position with hands placed shoulder width apart, having elbows flexed at a 90° angle, and pushing upward as a complete push-up) [33], with a greater number of 90° push-ups completed representing greater muscular endurance and strength. Flexibility was assessed by a modified sit and reach test (in inches) [34] and included mothers being in a seated position on the floor (back against the wall), with their legs straight and their hands extended out in front of them. Mothers were instructed to reach forward as far as they could with their hands over each other, over a three-foot ruler, without removing their body from the wall and holding this position for at least two seconds, with greater inches indicating greater flexibility.

#### Self-efficacy

Self-efficacy was assessed using two measures: The Barriers Self-Efficacy Scale [35] and the Self-Efficacy for Physical Performance Scale [36]. The 14-item Barriers Self-Efficacy Scale measures a mother’s level of confidence in engaging in PA despite barriers experienced over the next 3 months, with higher scores (range = 0–100) reflecting higher self-efficacy for PA in the face of barriers (*α range* = 0.88–0.95). The 5-item Self-Efficacy for Physical Performance Scale measures a participant’s self-efficacy to currently perform multiple activities that improve fitness (e.g., walking a mile, doing push-ups), with higher scores (range = 0–100) indicating higher self-efficacy for fitness (*α range* = 0.69-0.83).

#### Social support

Social support for PA was assessed using the 15-item Social Support for Exercise Survey (SSFE) [37]. The SSFE measures the degree of social support received from one’s family and friends to engage in PA, with higher scores reflecting more social support received from each of these support networks (*α range* = 0.85–0.95 for family subscale; *α range* = 0.87–0.93 for friends subscale).

#### Intervention acceptability

At 3 months post-intervention, mothers participated in a focus group (conducted separately for CBI and HBI participants) that was videotaped and transcribed to assess mothers’ experiences in their respective PA programs (i.e., PA barriers and facilitators) and recommendations for program improvement as indicators of intervention acceptability. Two open-ended questions were asked:

1. “What factors affected your PA goals and participation in your program?”
2. “If there was anything you could change in the program, what would it be?”

### Analyses

#### Power analysis

An a priori power analysis (using the G Power software) [38] indicated that 52 participants were needed to obtain statistical power to detect meaningful associations among study variables with a medium effect size (0.50) at the recommended 0.80 level. However, a previous study examining the effectiveness of a pilot PA intervention among multiethnic postpartum women reported a large effect size (Cohen’s *d* = 2.2) with a sample of 20 mothers [39]. Therefore, effect sizes for the current pilot study were reported to help inform future PA interventions with low-income, ethnic minority mothers.

#### Progression criteria

Progression criteria were set prior to study implementation in accordance with published guidelines set forth for studies assessing feasibility of physical activity interventions [40] to help identify whether aspects of the study would need to be amended before proceeding to a large-scale randomized control trial (RCT) trial. Criteria for the current study included: (A) recruiting a total sample size of 52 mothers; (B) having a retention rate of no less than 60% in each group at 3 months post-intervention; and (C) reporting of no serious adverse events, such as hospitalization, a life-threatening condition, death, and any adverse events associated with the intervention.

#### Quantitative Data

Pearson’s chi-squared and independent samples *t*-tests were conducted for categorical and continuous variables, respectively, to assess for between group differences (i.e., CBI and HBI groups) on study characteristics at baseline. Pearson product-moment correlations and repeated measures ANOVA analyses were used to identify possible covariates [i.e., ethnicity, number of children, body mass index, baseline fitness levels (based on cardiorespiratory fitness; VO_2max_)] on study outcomes. Only baseline fitness levels were significantly associated with PA and fitness outcomes and were included as a covariate in subsequent analyses. Repeated measures ANCOVA analyses with Greenhouse–Geisser corrections for violations of sphericity were conducted to test for possible group by time effects for randomization group (CBI vs. HBI) on PA (CALQ, FitBit), physical fitness (cardiorespiratory fitness, muscular endurance and strength, flexibility), self-efficacy, and social support across the four study time points (baseline, 1 month, 2 months, and 3 months), adjusting for baseline fitness levels. The effect sizes for time and group effects were presented as partial eta squared (η_p_^2^), as is recommended for mixed models [41]. The least-squares means method was used to compare group means for all significant effects. Intent-to-treat analyses were used to handle missing data for PA and fitness such that available data for a particular time point (e.g., baseline) was carried forward to replace missing data for a subsequent time point to take a more conservative data imputation approach of no change in the outcome variables for these participants over time.

#### Qualitative data

An inductive thematic analysis approach was used to analyze focus group transcripts [42]. Potential themes and subthemes were developed from marginal codes, which were used as a codebook. Two independent coders pilot tested the codebook on 50% of focus group transcripts. Further revisions to codes and definitions were made to accurately capture participants’ experiences. During the second phase of analyses, two independent coders were paired and utilized the codebook created in the first phase to apply codes to excerpts. Any disagreements on application of codes were resolved through a group discussion until consensus was reached. The last half of the coded transcripts were assessed for level of agreement in codes applied using Cohen’s kappa. Interrater reliability was strong (*k* = 0.80).

## Results

### Participant characteristics

Mothers in both the CBI and HBI groups were similar on all sociodemographic characteristics (see Table 2). On average, participants were 32 years of age (*SD* = 5.62, range = 25–46 years of age), had three children (*SD* = 1.47, range = 1–8 children), and had a body mass index of 32 (*SD* = 6.74, range = 20.47–50.32). A majority were English speakers (60%), Latina (59%), married or living with their partner (73%), were unemployed (72%), and had a total annual family income of less than $20,000 (57%). In terms of education, 43% had a high school education or less.

**Table 2:** Baseline sociodemographic characteristics by randomization group

	**CBI** *n* = 20	**HBI** *n* = 10
**Age** [*M* (*SE*, range)]	32.95 (± 5.99, 26–46)	31.20 (± 4.87, 25–42)
**Number of children** [*M* (*SE*, range)]	2.70 (± 1.63, 1–8)	2.50 (± 1.18, 1–5)
**Language** [*n* (%)]		
English	12 (60)	6 (60)
Spanish	8 (40)	4 (40)
**Ethnicity** [*n* (%)]		
Latina	12 (71)	4 (40)
African-American	2 (12)	2 (20)
Caucasian	0 (0)	3 (30)
Other	3 (18)	1 (10)
**Marital status** [*n* (%)]		
Single/not married	5 (25)	3 (30)
Married/living together	15 (75)	7 (70)
**Education** [*n* (%)]		
High school diploma/GED or less	9 (45)	4 (40)
Some college	6 (30)	4 (40)
College graduate or more	5 (25)	2 (20)
**Employment status** [*n* (%)]		
Employed	4 (21)	4 (40)
Unemployed	15 (79)	6 (60)
**Total annual family income** [*n* (%)]		
< $20,000	12 (60)	5 (50)
$20,000–$34,999	6 (30)	2 (20)
≥ $35,000	2 (10)	3 (30)

Note: Pearson’s *χ*^2^ and independent samples t-tests were conducted to examine sociodemographic differences by randomization group (CBI vs HBI). Results showed no significant differences by randomization group (*p* > .05)

### Study feasibility and progression criteria

**Fig. 2:**
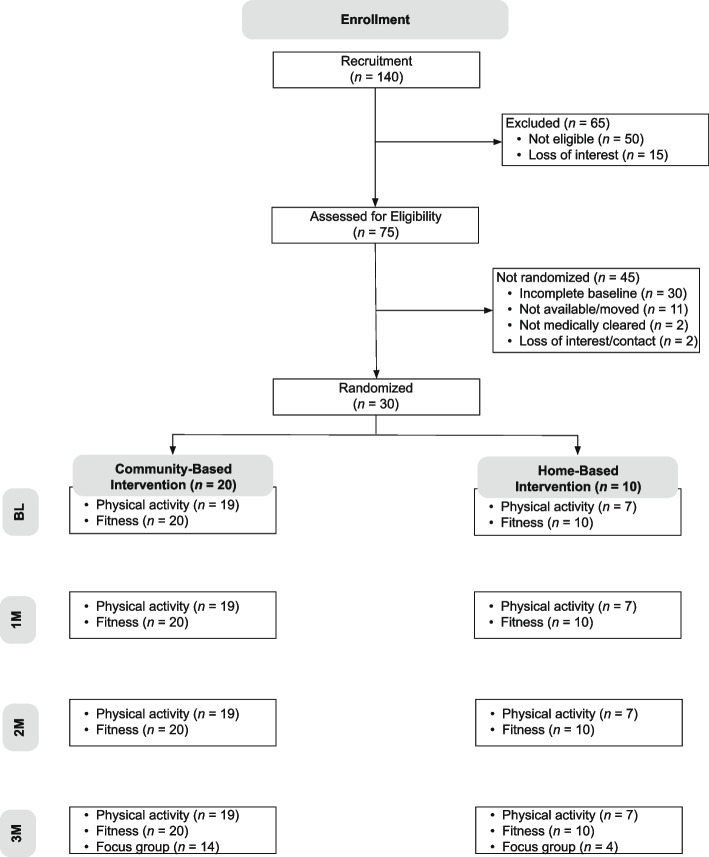
CONSORT statement

The Consolidated Standards of Reporting Trials (CONSORT) diagram of study recruitment, enrollment, and retention is provided in Fig. 2. Of 140 mothers recruited for the study, 50 were ineligible (40% not available to attend the PA classes, 34% not medically cleared to participate, 26% exercising more than 90 min/week), and 15 were no longer interested in participating or lost to contact. Of the remaining 75 mothers, 45 were not randomized (67% did not complete their baseline assessments and therefore were no longer eligible for the study, 24% were no longer available for the study/moved from the area, 5% were no longer medically cleared, and 4% were no longer interested in participating or lost to contact). The remaining 30 mothers were randomized to either a 3-month CBI (*n* = 20) or HBI (*n* = 10) PA program (a priori target goal was 52 mothers).

Follow-up assessments were also conducted for mothers randomized to the CBI and HBI groups with participant retention rates of 70%, 63%, and 70% for 1-month, 2-month, and 3-month study time points, respectively (a priori target goal was ≥ 60% retention). Retention rates by randomization group were similar across all time points (see Fig. 2). On average, CBI participants attended 7 out of 12 classes (58%), with 55% (*n* = 11) attending at least half of the classes (range = 1–12 classes). Of participants randomized to the HBI, 50% completed their PA logs at all four time points. A total of 4 of the 10 mothers in the HBI group (40%) and 14 of 20 mothers in the CBI group (70%) participated in the 3-month post-intervention focus group. No adverse events were reported for mothers in either the CBI or HBI groups throughout their study participation (a priori target goal was 0%).

### Change in PA and fitness over time

**Fig. 3:**
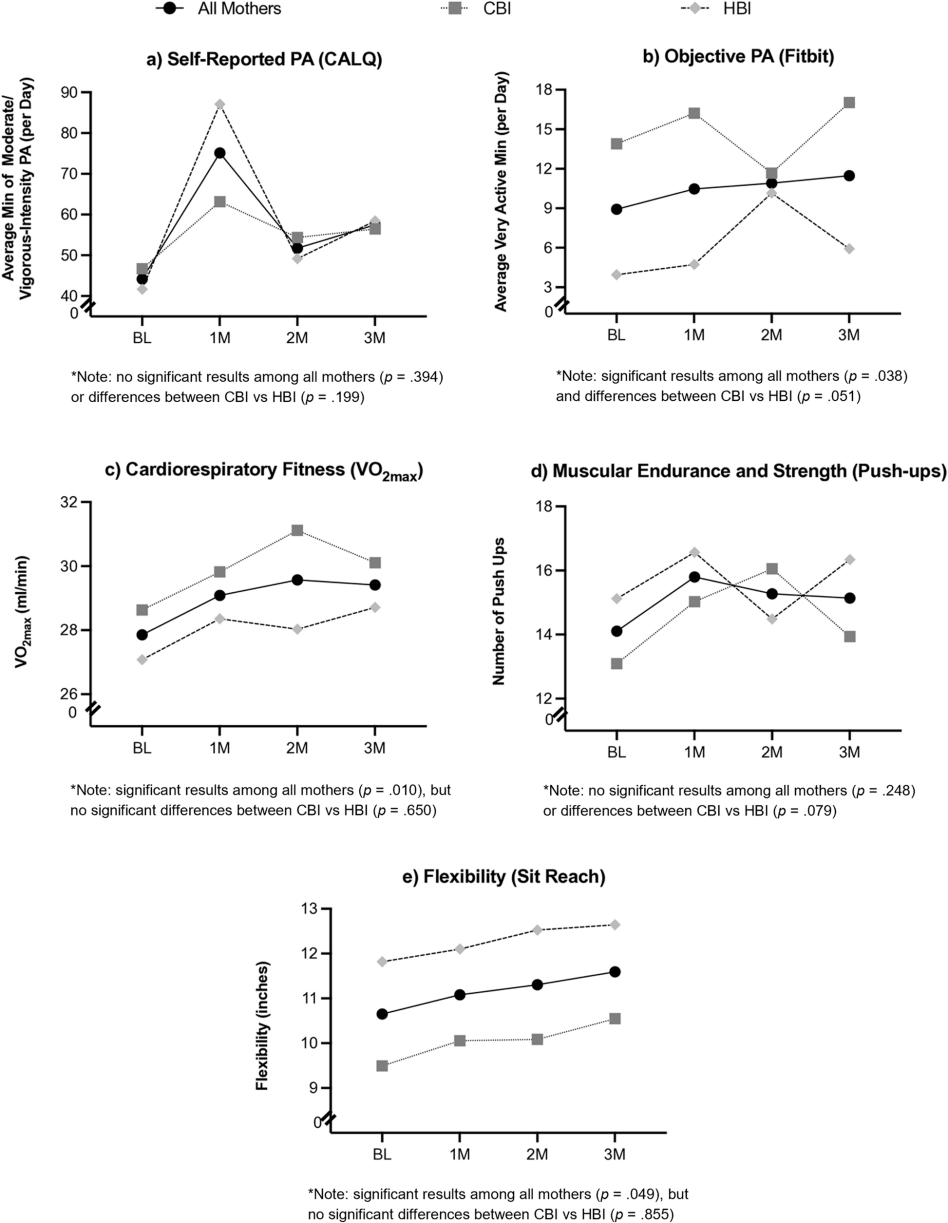
Change in physical activity and fitness levels over time and by randomization group

Approximately 69% (*n* = 20) of mothers met ACSM PA recommendations to engage in 30 min or more of moderate physical activity per day (based on self-reported PA) at 3 months post-intervention regardless of group assignment. All mothers, controlling for baseline fitness levels, showed a significant increase in objective PA [Fitbit; *F*(1, 21) = 4.91, *p* = . 038, *η*_*p*_^*2*^ = 0.19 (linear pattern)], cardiorespiratory fitness [VO_2max_;* F*(1, 25) = 7.84, *p* = 0.010, *η*_*p*_^*2*^ = 0.29 (linear pattern)], and flexibility [Sit & Reach; *F*(1, 25) = 4.28, *p* = . 049, *η*_*p*_^*2*^ = . 15 (linear pattern)] from baseline to 3 months post-intervention (see Fig. 3a–e). There were no significant changes in self-reported PA [CALQ; *F*(2.02, 48.52) = 0.95; *p* = . 394, *η*_*p*_^*2*^ = . 04] or muscular endurance and strength [Push-ups; *F*(3, 75) = 1.40; *p* = 0.248, *η*_*p*_^*2*^ = 0.05] over time.

### Group differences in PA and fitness over time

Mothers in the CBI group had higher objective PA levels throughout all time points compared to mothers in the HBI group [Fitbit; *F*(1, 21) = 4.30, *p* = 0.051, *η*_*p*_^*2*^ = 0.17 (cubic pattern); see Fig. 3b]. There was also a marginally significant trend in which mothers in the HBI group had greater muscular endurance and strength at 3 months post-intervention compared to mothers in the CBI group [Push-ups; *F*(1, 25) = 3.36, *p* = 0.079, *η*_*p*_^*2*^ = 0.12 (quadratic pattern); see Fig. 3d]. There were no significant group differences in self-reported PA [CALQ; *F*(2.02, 48.52) = 1.67, *p* = 0.199, *η*_*p*_^*2*^ = 0.07], cardiorespiratory fitness [VO_2max_; *F*(2.32, 57.94) = 0.48, *p* = 0.650, *η*_*p*_^*2*^ = 0.02], or flexibility [Sit & Reach; *F*(2.13, 53.25) = 0.17; *p* = 0.855, *η*_*p*_^*2*^ = 0.01] over time.

### Group differences in self-efficacy over time

**Fig. 4:**
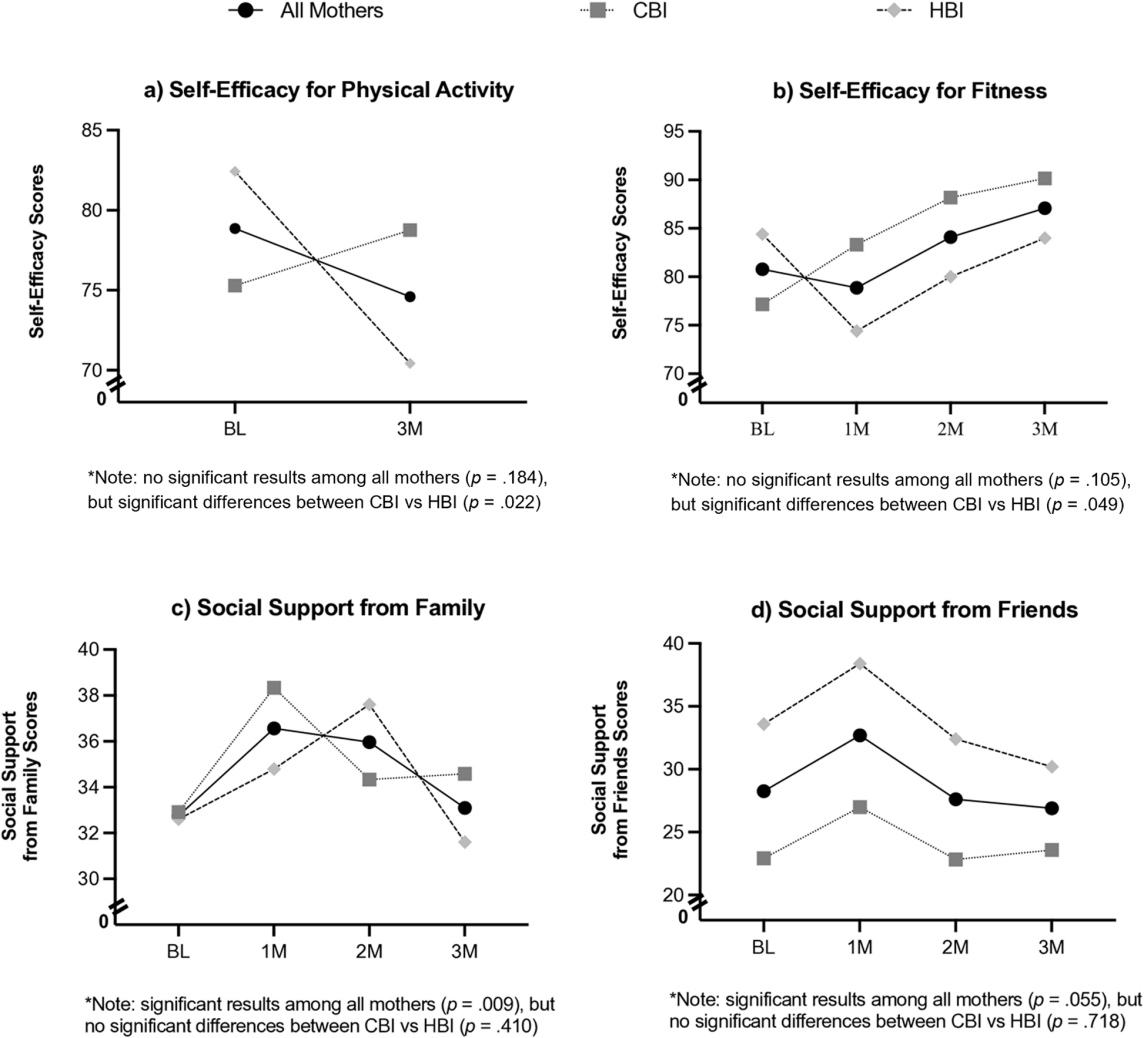
Changes in psychosocial outcomes (self-efficacy for physical activity and fitness; social support from family and friends) over time and by randomization group

Collectively, mothers showed no significant changes in self-efficacy for PA over time [*F*(1, 18) = 1.91, *p* = 0.184, *η*_*p*_^*2*^ = 0.10]. However, there were significant differences in self-efficacy by randomization group over time. Specifically, mothers in the CBI group had higher self-efficacy for PA at 3 months post-intervention than mothers in the HBI group [*F*(1, 18) = 6.28, *p* = 0.022, *η*_*p*_^*2*^ = 0.26 (linear pattern)], who showed decreased self-efficacy over time (see Fig. 4a).

Furthermore, mothers showed no significant changes in self-efficacy for fitness over time [*F*(2.09, 31.39) = 2.40, *p* = 0.105, *η*_*p*_^*2*^ = 0.14]. However, there were significant differences by randomization group. Specifically, mothers in the CBI group demonstrated steady increases in their self-efficacy for fitness over time compared to mothers in the HBI group who showed no changes over time [*F*(1, 15) = 4.57, *p* = 0.049, *η*_*p*_^*2*^ = 0.23 (quadratic pattern); see Fig. 4b].

### Group differences in social support over time

All mothers showed significant changes in social support from family over time [*F*(1, 15) = 8.85, *p* = 0.009, *η*_*p*_^*2*^ = 0.37 (quadratic pattern)]. Specifically, social support from family increased from baseline to 1 month, then decreased from 1 to 3 months post-intervention (see Fig. 4c). Similarly, all mothers showed a marginally significant change in social support from friends over time such that social support increased from baseline to 1 month, then decreased from 1 to 3 months post-intervention [*F*(1, 15) = 4.35, *p* = 0.055, *η*_*p*_^*2*^ = 0.23 (cubic pattern); see Fig. 4d]. No significant randomization group differences in social support from family [*F*(3, 45) = 0.98, *p* = 0.410, *η*_*p*_^*2*^ = 0.06] or friends [*F*(1.73, 25.91) = 0.29, *p* = 0.718, *η*_*p*_^*2*^ = 0.02] were found over time.

### Qualitative results

Qualitative data from five focus groups with 18 mothers (14 CBI, 4 HBI) resulted in four main themes: barriers to PA (3 subthemes), facilitators to PA (2 subthemes), program benefits (0 subthemes), and future program suggestions (2 subthemes). The definition of each main theme and subtheme, as well as the number of instances in which the theme/subtheme was discussed by mothers are presented in Table 3.

**Table 3:** Qualitative themes for barriers/facilitators to physical activity, program benefits, and future program suggestions

**Main theme**	*n*^a^	Definition
**Barriers to physical activity**	6	Participants mentioned various obstacles as barriers to physical activity.
Relationship with others	3	Participants mentioned demands of family, friends, and responsibilities surrounding children as a barrier to exercise, and reasons for not putting themselves first.
Self-doubt	2	Participants mentioned not believing in themselves as a barrier to physical activity.
**Facilitators to physical activity**	50	Participants mentioned family/friends, group facilitators/intervention as a whole, and other people inspiring them to engage in physical activity.
Social support	46	Participants mentioned family/friends, group facilitators/intervention as a whole, and other people inspiring them to exercise, includes receiving and giving social support to exercise.
Program materials/facility	4	Participants mentioned that educational materials/class content, devices, and the facility where the intervention was held as a facilitator to exercise.
**Program benefits**	41	Participants mentioned improvements in health outcomes/behaviors/mindset, use of self-care for themselves as a program benefit.
**Future program suggestions**	39	Participants mentioned several program suggestions that may be implemented in the future.
Family participation	8	Participants expressed their opinions in involving or incorporating family into the program.
Program structure	31	Participants mentioned suggestions/changes surrounding the structure of the program.

^a^Number of instances code was discussed by participants

#### Barriers to PA

Mothers in the CBI, but not the HBI, discussed various barriers to PA while participating in their program, including relationships with others, self-doubt, and household chores. For “*relationship with others*,” children were described as a barrier to PA. An association of guilt seemed to overcome the mothers who felt they could not allot time to exercise because of their child wanting to spend time with them.

“I could lock myself in the room and workout in there. But I can’t even work out comfortable because my daughter’s knocking at the door and crying ‘come out, come out’. It’s like ‘oh my God am I going to work out and hear you crying?’…It’s a huge guilt factor…cuz [my daughter] wants you there all the time because you’re home.”

Mothers in the CBI also expressed having self-doubt in their ability to engage in PA due to a fear of engaging in PA alone and feelings of being easily discouraged.

“Before this program we [moms] were, especially me I was discouraged...If one day I exercised, the next day I was like, “Okay, I don’t know if this is going to work out.”

#### Facilitators to physical activity

Mothers discussed the importance of social support from family and friends, along with program materials, as facilitators to PA. Specifically, seven mothers in the CBI group, compared to one mother in the HBI group, discussed the imperative role that their husbands played in supporting their exercise goals by taking over childcare duties, which allowed mothers to set aside time to exercise. CBI mothers also described their husbands as being encouraging and even joining them in walking as a form of social support.

“He’s helping more with the kids and my husband is taking care of them while I go to the gym if I’m gonna do Active Moms...and he’s actually encouraging it.” “My husband has always motivated me and now he supports me. He takes me to the park and he tells me come on go and walk with him and the kids.”

One mother from the HBI discussed the improvement in the relationship with her husband as he saw her engage in PA.

“Yes, because when you’re motivated, for example in my case when my husband sees me happy, he sees me active that I do things and he too is happy, and oh you finished early and let’s go I’ll invite you to dinner or something and there’s a better relationship of course.”

Mothers in both groups also discussed their children as inspiration and motivation to engage in exercise. Mothers pursued a healthier lifestyle through PA to engage in more recreational activities with their children, serve as a healthy role model for them, and live longer for their children. Increased participation in recreational activities was also reported to have subsequently improved their relationship with their children. One mother from the HBI group discussed the motivation her children instilled in her when they engaged in PA together.

“For my children, if they see me go out to walk, they put on tennis shoes…it makes me happy that they say, ‘oh we’re going to walk with my mom’ and my daughter says, ‘oh you’re gonna do Active Moms.’ It also gives me motivation that they [children] are also involved, that they know what I do and why I do it [exercise].”

One mother from the CBI group discussed her children’s role in her PA maintenance.

“For me I thought, ’Oh no, how am I going to do it.’ But so far it has worked out and I like it. It’s a good thing because I’m getting older, so it’s good that I’m getting it now and it’s going to continue because I have to stick around for my kids.”

#### Program benefits

A total of 16 out of 18 mothers (89%) reported experiencing health benefits as a result of their program participation. Three prominent topics emerged, including stress management, self-confidence, and prioritizing self-care. Mothers in the CBI discussed how their stress levels decreased, which improved their sleep and mood. They also discussed an increase in their overall self-confidence to engage in PA that was also noticed by their family members and friends.

“Now [after the intervention] it’s like I’m more active, less stressed. I hang out with her [my daughter]. I play with her a lot. I have a lot of energy. I started taking care of myself, liking myself, and telling myself that it’s not impossible.”

One mother from the HBI group discussed the effects of PA on her overall well-being and outlook on life.

“When you are good, you feel good and you reflect well. Sometimes when you are at home you think a lot of dumb things but when you go out to walk your mind clears... You analyze the bad and the good and the good is how when you’re exercising you are generating good health for your body and your emotional health too.”

Mothers from both groups discussed the importance of self-care and prioritizing themselves more after participating in their program. Specifically, one mother from the HBI group recalled her ability to bypass her husband's discouraging comments regarding her weight and her decision to continue to engage in PA because of the changes she saw in herself.

“I did notice that my husband would tell me why do you do that if you’re not losing weight and I would think ‘yes that’s true, I’m not noticing anything in my physical appearance’. But after I began to analyze [and think], it’s more of making a habit and as the habit is made, one moderates their lifestyle. And I said, well maybe physically I’m not looking different, but I am feeling different.”

Furthermore, a mother from the CBI group explained the importance of setting aside time from other priorities, such as chores, to engage in PA to live a healthier lifestyle.

“I think this is an excellent program and, you know, moms should start thinking about themselves because chores are always going to wait, and if you don’t think about yourself then nobody else is. I think this has helped me a lot. I’m really thankful for coming here and meeting the other moms.”

#### Future program suggestions

Mothers provided suggestions for future PA programs related to family participation and program structure. For family participation, most mothers in the CBI indicated that the program worked best without including their spouses because it offered them alone time that they otherwise would not normally have.

“I feel that when I come here or when I go to the gym or I go for a walk I could join in with my family but there are times that you want that one hour to yourself where you’re not, ‘oh what are you going to cook,’ ‘oh you need to do this,’ ‘oh, you know mommy...’ You know, all the multi-tasking and for one hour you get to break free.”

Mothers in the CBI and HBI also advocated for graduates of the program to return to serve as group facilitators for future classes and discussed the various benefits of having other “successful” mothers lead the class, including maintaining their own PA gains.

“I think [bringing graduates of the program] would be a good thing. When we [mothers] share here, we all have to know everybody’s obstacles and challenges. I think sometimes when you hear somebody’s story, it might encourage you.” “Even if we helped the next cohort, they probably could help us for accountability…cuz they can help us as much as we’re helping them.”

Additionally, mothers from the CBI discussed the need to increase the length of the program and to transition from the group-based format to the home-based format at a slower pace with more group instruction days.

“I would suggest two things, that it would be longer and more days. Three months is not a lot because one needs more time. That there would be more days because two [days per week] were not enough, if it could be all week up to 6 months is good.”

Finally, two mothers from the HBI discussed their preference for group-based PA.

“In my case, I would have liked to be in the group with the other people more than being alone because when someone has little willpower, being with other people motivate[s] you a lot. A while back I was in another program to lose weight and it was very important to be with other people because you learn directly from other people, what they think and how they make things work, so I do think being with other people helps you a lot.”

## Discussion

The primary aim of this pilot study was to assess the feasibility (i.e., recruitment, retention, and participant safety) of delivering a study protocol of a 3-month CBI and a HBI among low-income, ethnic minority mothers. Results demonstrated that the study protocol met two out of three a priori progression criteria (> 60% retention rate, 0% adverse events) but did not meet the goal of recruiting and randomizing at least 52 mothers into the study (current sample size was 30 mothers). Of 75 mothers who were identified to be eligible for the study, 45 (60%) were not randomized largely due to not completing their baseline PA/fitness assessments or no longer being available to participate. These results provide valuable data for researchers to consider when designing their enrollment protocols for larger RCTs among low-income, ethnic minority mothers. Specifically, protocols that account for competing time demands and maternal stressors (e.g., heavy childrearing responsibilities, financial strain in caring for children) are essential to address these common barriers to exercise participation, as previous studies have shown low-income, ethnic minority mothers who experience more frequent maternal stressors are less likely to attend PA programs and are less likely to demonstrate PA gains, despite being a population most in need of these programs [9].

This study also explored the impact that the CBI and HBI had on PA and fitness levels, self-efficacy, and social support in this population. Results showed that mothers in both the CBI and HBI significantly increased their objective PA levels (i.e., Fitbit), cardiorespiratory fitness, and flexibility over time. These results are consistent with a CBI study that transitioned mothers from a 4-week group-based to a 4-week home-based format (based on social cognitive theory) and showed significant improvements in PA during the group-based phase while maintaining these changes during the home-based phase [17]. Collectively, these results provide support for the effectiveness of social cognitive theory-based strategies for improving PA levels using both CBI and HBI formats. Additional studies are needed to identify specific components of social cognitive theory that may be particularly helpful in improving PA and fitness levels among low-income, ethnic minority mothers.

Our results also showed that mothers randomized to the CBI showed greater improvements in self-efficacy for PA and fitness compared to mothers in the HBI. These results are consistent with two previous studies that demonstrated an increase in self-efficacy for PA among mothers participating in a 1- to 2-month CBI [17] and are possibly a result of CBI mothers receiving initial support and strategies to overcome PA barriers from other mothers during the group-based phase and then transitioning to a home-based phase where they became more independent exercising on their own. In contrast, mothers in the HBI did not have the opportunity to participate in group discussions with other mothers, which may explain why their self-efficacy levels declined. During the focus groups, mothers in the CBI also shared that receiving compliments by their family and friends on their physical appearance boosted their self-efficacy and motivation for engaging in PA. Interestingly, their self-efficacy levels continued to improve at 3-months post-intervention, even as they transitioned to exercising on their own. Future studies are needed to examine the long-term effects of increased self-efficacy levels on sustaining PA and fitness gains in this population.

Although mothers in both the CBI and HBI demonstrated 1-month increases in social support for PA from their family and friends, their social support decreased over the subsequent 2 months. For mothers in the CBI, the 1-month improvements in their social support may be due to the staged design of the CBI where they initially attended two group exercise sessions per week. However, these group exercise sessions dropped to once per week during the second month, and they exercised completely on their own in the last month. Therefore, they may have experienced less support as the number of group exercise sessions decreased over time. Similarly, in the focus groups, CBI mothers discussed initially receiving social support from their spouses in the form of helping with childcare, which improved their overall relationship. Their spouses also provided words of motivation as they began their exercise program. However, it may be that they received less support from their spouses over time. These results extend the findings of a recent systematic review which found that spousal support can serve as a barrier or facilitator to PA depending on their spouses’ willingness to share childcare/household responsibilities [5]. In contrast, HBI mothers discussed heavily relying on support from their children to engage in PA, as they received little support from their spouse or friends. Despite the lack of motivation received from their spouses, mothers in the HBI seemed to have continued to follow the intervention due to the motivation they received from their children and the overall changes they saw in themselves. These results are consistent with other studies showing how mothers’ desire to be a role model for their children increase their self-efficacy for PA [5]. Given these results, future PA programs for mothers might benefit from incorporating their children or other sources of support to promote PA and fitness levels over time.

## Study limitations

Our results should be interpreted with caution given several limitations. First, this study included a relatively small sample size (*n* = 30). However, our results showed medium to large effect sizes (*η*_*p*_^*2*^ = 0.15–0.38) when examining the impact of the CBI and HBI on post-intervention PA and fitness outcomes, which is consistent with the effect sizes found in previous PA studies with mothers [39]. Nevertheless, additional studies in this research area, with larger sample sizes and a control group (no treatment), would aid in generalizing and supporting our findings. Such studies would also help identify potential mediators (e.g., self-efficacy, social support) and moderators (e.g., number of children, marital status) that may influence intervention effects on PA and fitness levels among low-income ethnic minority mothers. Third, the 3-month intervention period, while useful for establishing initial intervention efficacy, is too short of a time-period to understand the sustained impacts of the interventions on PA and fitness levels. Longer intervention periods are indicated to assess such effects more thoroughly. Finally, given the time period at which these programs were offered (2012–2014), additional studies are needed to explore newly available technologies that promote PA in low-income, ethnic minority populations and that address COVID-related barriers to participation in such programs.

## Conclusions

The current results demonstrate the effectiveness of both CBIs and HBIs for improving PA and fitness levels among low-income, ethnic-minority mothers. Additionally, the CBI was most effective in increasing self-efficacy for both PA and fitness relative to the HBI. These results have important implications for improving access to health promotion programs in low-income communities. Specifically, implementing these interventions in the community (CBI) or at the convenience of the mother’s home (HBI) provide more options to support low-income mothers in their efforts to engage in PA. These manualized CBI and HBI formats also provide viable alternatives to going to a gym for meeting PA goals by addressing the many challenges associated with motherhood (e.g., lack of support, balancing self-care with caregiving responsibilities) and can be delivered by health care professionals and paraprofessionals (e.g., community health workers). Specifically, future interventions should consider incorporating graduated participants from previous cohorts as facilitators for new participants. This modification in the protocol may engage more mothers to participate in PA programs, as group facilitators with similar backgrounds and experiences as mothers can help participants problem-solve to enroll in such studies. These protocol modifications could then lead to the implementation of larger randomized controlled studies to demonstrate the potential longer-term health benefits of CBIs and HBIs, as well as to identify the mechanisms by which these interventions lead to optimal health outcomes in this population. Such interventions should be tailored to the cultural resources, strengths, and community resources that low-income, ethnic-minority mothers, and their families have to adopt and maintain health behaviors. Finally, further research is needed to identify intervention components, designs, and delivery modalities (e.g., promotora-led programs) that may be most effective in promoting PA and reducing health disparities in this and similar populations.

## Data Availability

The data that support the findings of this study are available from the corresponding author [GU] upon reasonable request.

## Abbreviations

CALQ: Check and line questionnaire
CBI: Community-based intervention
CONSORT: Consolidated Standards of Reporting
HBI: Home-based intervention
PA: Physical activity
SSFE: Social support for exercise
